# Synergy of Tumor Microenvironment Remodeling and Autophagy Inhibition to Sensitize Radiation for Bladder Cancer Treatment

**DOI:** 10.7150/thno.45358

**Published:** 2020-06-19

**Authors:** Tingsheng Lin, Qing Zhang, Ahu Yuan, Baojun Wang, Feifei Zhang, Yuanzhen Ding, Wenmin Cao, Wei Chen, Hongqian Guo

**Affiliations:** 1 Department of Urology, Drum Tower Hospital, Medical school of Nanjing University, Institute of Urology, Nanjing University, Nanjing 210008, Jiangsu, China.; 2 State Key Laboratory of Pharmaceutical Biotechnology, Medical School of Nanjing University, Nanjing 210093, Jiangsu, China.

**Keywords:** Tumor microenvironment, manganese dioxide, autophagy inhibitor, radiation therapy

## Abstract

Tumor hypoxia, acidosis, and excessive reactive oxygen species (ROS) were the main characteristics of the bladder tumor microenvironment (TME), and abnormal TME led to autophagy activation, which facilitated cancer cell proliferation. The therapeutic efficacy of autophagy inhibitors might also be impeded by abnormal TME. To address these issues, we proposed a new strategy that utilized manganese dioxide (MnO_2_) nanoparticles to optimize the abnormal TME and revitalize autophagy inhibitors, and both oxygenation and autophagy inhibition may sensitize the tumor cells to radiation therapy.

**Methods:** By taking advantage of the strong affinity between negatively charged MnO_2_ and positively charged chloroquine (CQ), the nanoparticles were fabricated by integrating MnO_2_ and CQ in human serum albumin (HSA)-based nanoplatform (HSA-MnO_2_-CQ NPs).

**Results:** HSA-MnO_2_-CQ NPs NPs efficiently generated O_2_ and increased pH *in vitro* after reaction with H^+^/H_2_O_2_ and then released the encapsulated CQ in a H^+^/H_2_O_2_ concentration-dependent manner. The NPs restored the autophagy-inhibiting activity of chloroquine in acidic conditions by increasing its intracellular uptake, and markedly blocked hypoxia-induced autophagic flux. *In vivo* studies showed the NPs improved pharmacokinetic behavior of chloroquine and effectively accumulated in tumor tissues. The NPs exhibited significantly decreased tumor hypoxia areas and increased tumor pH, and had remarkable autophagy inhibition efficacy on bladder tumors. Finally, a significant anti-tumor effect achieved by the enhanced autophagy inhibition and radiation sensitization.

**Conclusions:** HSA-MnO_2_-CQ NPs synergistically regulated the abnormal TME and inhibited autophagic flux, and effectively sensitized radiation therapy to treat bladder cancers.

## Introduction

Autophagy is a catabolic process that degrades and recycles proteins and organelles to produce amino acids and nucleotides to support cancer cells 1,2. It plays a critical role in tumor progression, metastasis, and resistance to multiple treatments 3-5. Hypoxia, acidic pH, and high ROS are the main characteristics of the tumor microenvironment (TME) and also stimulate tumor autophagy. For instance, Yu-Long demonstrated that autophagy was activated by hypoxic stress in the TME via the hypoxia-inducible factor-1α (HIF-1α)/AMPK pathway, which promoted glioblastoma cell survival 6. Maria reported that the acidic microenvironment also upregulated autophagy in melanoma and promoted tumor progression 7. Therefore, regulating an aberrant TME could probably suppress tumor autophagy and potentiate multiple tumor treatments.

Previous studies have tried to inhibit tumor autophagy by regulating TME or introducing an autophagy inhibitor. Noman *et al* have shown that oxygen supply could relieve tumor hypoxia and inhibit hypoxia-induced autophagy 8. Tumor autophagy could also be blocked by systemic treatment with sodium bicarbonate, which increased intratumoral pH 9. On the other hand, some other researchers utilized an autophagy inhibitor, including chloroquine (CQ), to block autophagic flux for tumor suppression 10. However, an acidic extracellular pH microenvironment significantly inhibited the therapeutic efficacy of chloroquine 11. Specifically, Pellegrini demonstrated that an acidic extracellular pH protonized nitrogenous organic compounds (chloroquine *et. al*) and induced inefficient cellular internalization 12. Based on the above findings, the conjunctive use of modulating TME (especially acidic and hypoxic microenvironments) and autophagy inhibitors could be more effective for inhibiting autophagy.

Recently, we found that manganese dioxide nanoparticles (MnO_2_) were a safe and effective nano-agent that could improve a hypoxic and acidic tumor microenvironment 13-15. MnO_2_ efficiently reacted to the excessive H_2_O_2_ and H^+^ in the TME and generated oxygen to modulate hypoxic, acidic, and high-ROS tumor microenvironments 16-20. Therefore, we wanted to explore the possibility of MnO_2_ as an autophagy inhibition. Furthermore, our previous study demonstrated that MnO_2_ nanoparticles acted as nanocarriers for *in vivo* delivery of various cationic drugs. Chloroquine is a cationic aminoquinoline, which could be used to prevent and treat malaria 21. Therefore, we speculated that chloroquine could be encapsulated in MnO_2_ nanoparticles to synergistically regulate the tumor microenvironment and block autophagic flux.

Albumin is the most abundant plasma protein, and has been extensively explored as a drug carrier due to its excellent biocompatibility. In this study, human serum albumin (HSA) acted as a reducing agent for KMnO_4_ and the template for MnO_2_ deposition to form the nanoparticles. During deposition, positively charged CQ was absorbed and entrapped in MnO_2_ to acquire HSA-MnO_2_-CQ nanoparticles (HMCQ NPs). Then, we used bladder cancer as a tumor model, which was one of the most common urologic cancers with a hypoxic and acidic microenvironment 22-24. After administration, the established nanomedicine HMCQ NPs sequentially decomposed in the TME to relieve hypoxia, neutralize hydrogen ions and alleviate the acidic microenvironment. Then, the encapsulated autophagic flux inhibitor chloroquine was gradually released and internalized into tumor cells in the neutral microenvironment. Additionally, the generated oxygen immobilized DNA damage to sensitize radiation therapy. There is ever-increasing preclinical and clinical evidence describing that inhibiting autophagy might be able to enhance the therapeutic efficacy of radiation 25-28. Radiation therapy also played an important role in bladder preservation therapy for bladder cancer, and some studies showed that the 5-year overall survival after radiation therapy was close to the standard radical cystectomy 29,30. Therefore, we combined HMCQ NPs and mediated autophagy inhibition with X-ray radiation for bladder cancer treatment. Finally, *in vitro* and *in vivo* studies showed that HMCQ NPs could modulate the aberrant tumor microenvironment, restore the autophagy-inhibiting activity of chloroquine and greatly enhance the therapeutic effect of radiation, which presented a promising strategy for bladder cancer treatment.

## Results and Discussions

### Preparation and Characterization of HSA-MnO_2_-CQ NPs

**Scheme 1:**
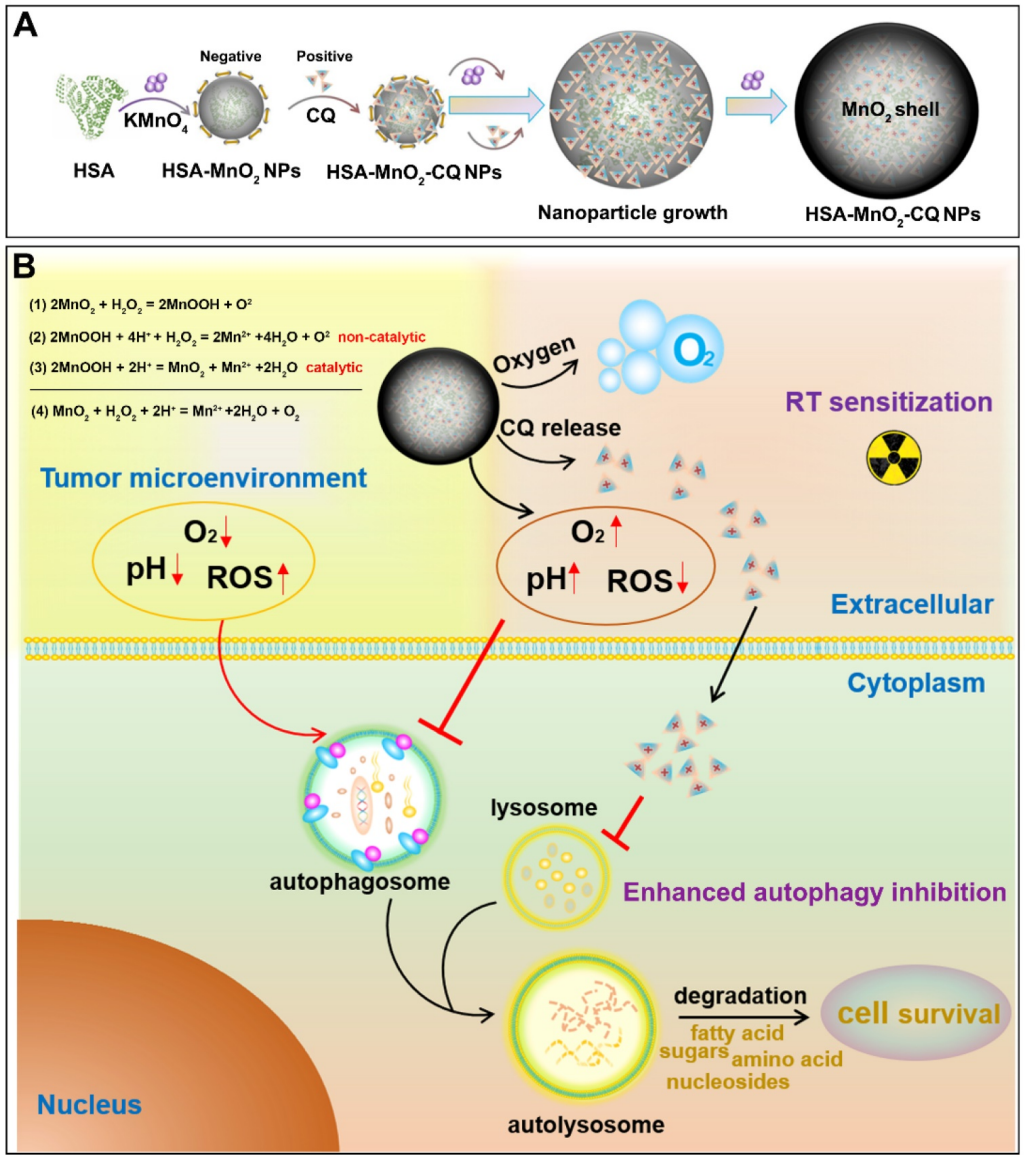
(A) Schematic illustration of the preparation of HSA-MnO_2_-CQ NPs. (B) The mechanism of HSA-MnO_2_-CQ NPs for enhancing the anti-tumor effect *by enhancing* the effects of autophagy inhibition and radiation by modulating tumor hypoxia and the acidic microenvironment.

**Figure 1:**
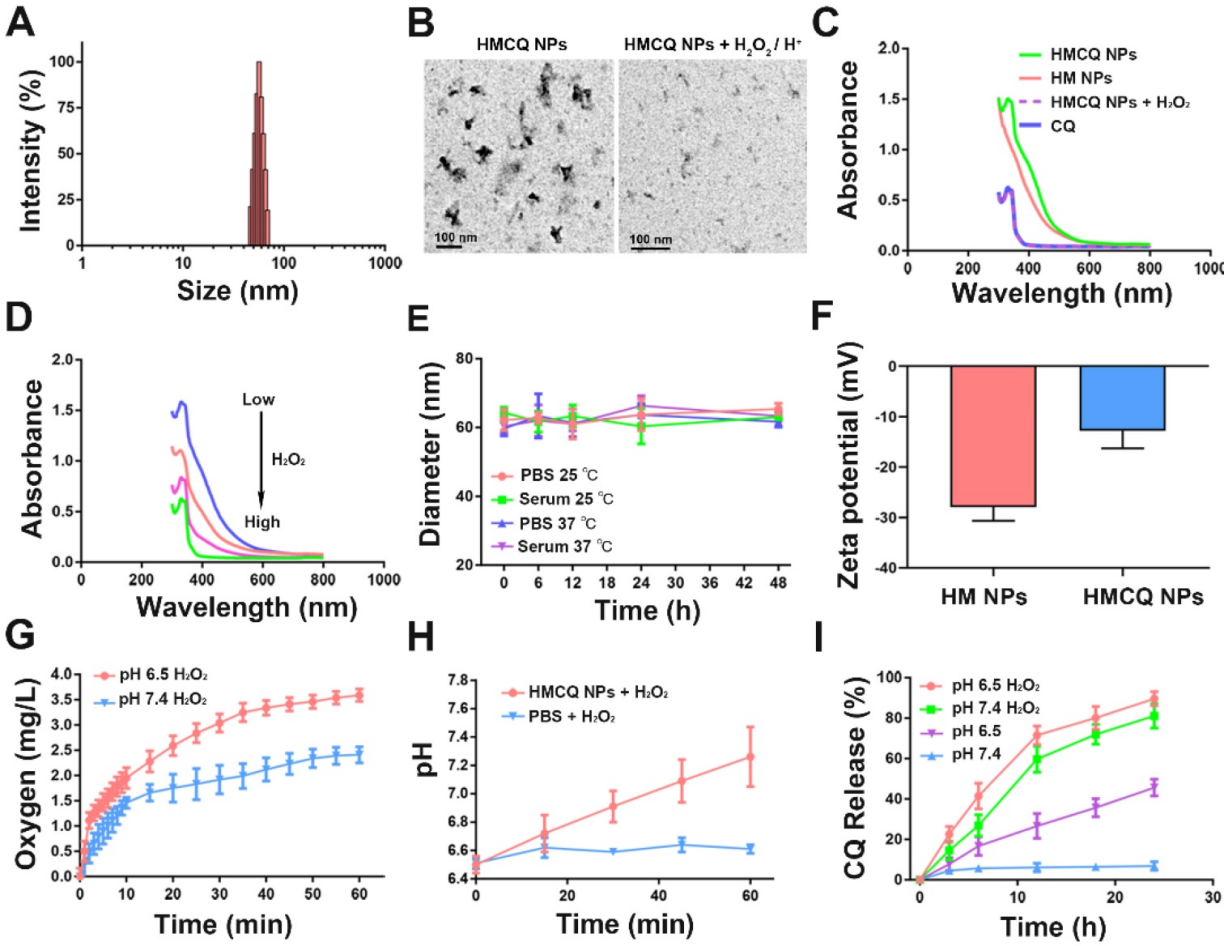
The main characteristics of HSA-MnO_2_-CQ NPs. (A) Size distribution of the HSA-MnO_2_-CQ NPs by DLS. (B) TEM images of the HSA-MnO_2_-CQ NPs and HSA-MnO_2_-CQ NPs reacted with H_2_O_2_/H^+^. (C) UV-absorbance spectrum of HSA-MnO_2_-CQ NPs, HSA-MnO_2_ NPs, free CQ, and HSA-MnO_2_-CQ NPs reacted with H_2_O_2_. (D) UV-absorbance spectrum of HSA-MnO_2_-CQ NPs reacted with various concentrations of H_2_O_2_. (E) DLS data of HSA-MnO_2_-CQ NPs incubated with PBS or serum at 25ºC or 37ºC, respectively. (F) Zeta potential of HSA-MnO_2_-CQ NPs and HSA-MnO_2_ NPs, (G) O_2_ generation at different pH values (6.5 and 7.4) from H_2_O_2_ solutions with HSA-MnO_2_-CQ NPs. (H) The increase in pH in HSA-MnO_2_-CQ NPs reacted with H_2_O_2_ solutions. (I) *In vitro* release of HSA-MnO_2_-CQ NPs in the presence or absence of H_2_O_2_ in PBS at a pH of 7.4 or a pH of 6.5, respectively. Data are shown as the mean ± SD.

The synthesis of HSA-MnO_2_-CQ NPs (HMCQ NPs) is illustrated in Scheme 1. HSA-MnO_2_ NPs (HM NPs) were first obtained *via* reduction of KMnO_4_ and deposition on albumin molecules. Our previous report showed that MnO_2_ nanoparticles were negatively charged and exhibited strong affinity with positively charged heterocyclic aromatic compounds (chloroquine *et. al*) in aqueous solution 31. To obtain HSA-MnO_2_-CQ NPs, chloroquine was gradually deposited when HSA-MnO_2_ formed. As shown in Figure S1C, the average diameter of HSA-MnO_2_ nanoparticles was 8.19 nm with a zeta potential of -27.94 mV. The unabsorbed CQ was then removed with ultrafiltration. Then, the size of the final HSA-MnO_2_-CQ NPs was 55.35 nm with a zeta potential of -12.80 mV (Figure 1A and 1F, Figure S1C). TEM also showed nanoparticles of similar size with good mono-dispersity (Figure 1B). The mechanism for forming HSA-MnO_2_-CQ nanoparticles was due to the interaction force between the cationic CQ (Figure S1A) and MnO_2_ including electrovalent and hydrogen bonds. The X-ray diffraction (XRD) profile of the HSA-MnO_2_ NPs or HSA-MnO_2_-CQ NPs exhibited a distinct peak at 2θ = 21.06°, which was in line with characteristic MnO_2_ peaks (Figure S1D). Finally, the HPLC profile of chloroquine extracted from HSA-MnO_2_-CQ NPs showed a similar mean retention time as free chloroquine standards, indicating successful encapsulation of CQ in the nanoplatform (Figure S1B). The status of the encapsulated CQ was further studied with X-ray diffraction (XRD). The results showed that the diffraction pattern of pure CQ exhibited several sharp peaks at 10.95º, 11.68º, 16.78º, 18.41º, 19.36º, 22.01º, 24.11º and 26.03º and a mixture of MnO_2_ nanoparticles and CQ. However, there was no characteristic peak on the XRD spectrum for HMCQ NPs, which indicated that CQ was randomly dispersed in the NPs in an amorphous state (Figure S1D).

When compared with free chloroquine, HSA-MnO_2_-CQ NPs showed a new UV-visible absorption band approximately 300~400 nm, which could be attributed to the surface plasmon band of MnO_2_. Then, the UV absorption of HSA-MnO_2_-CQ NPs at 300~400 nm gradually decreased with an increased concentration of H_2_O_2_, whereas the characteristic peak of chloroquine at 334 nm remained constant (Figure 1C and 1D). TEM images also showed that the nanoparticles were degraded after reacting with H_2_O_2_/H^+^ (Figure 1B). The size of HSA-MnO_2_-CQ NPs in PBS/serum at 37 °C or 25 °C remained stable (Figure 1E), potentially indicating excellent stability *in vivo*.

Then, we investigated the ability of the HSA-MnO_2_-CQ NPs to generate O_2_ and neutralize hydrogen ions *in vitro*. As shown in Figure 1G, O_2_ was efficiently produced when it reacted with H_2_O_2_, and the acidic pH promoted O_2_ generation. Besides the production of O_2_, the reaction also caused an increase in the pH of the reaction medium via consumption of H^+^ ions (Figure 1H). Specifically, a significant increase in the PBS pH from 6.5 to 7.2 was observed during the reaction of HSA-MnO_2_-CQ NPs with H_2_O_2_. The ability of HSA-MnO_2_-CQ NPs to consume H^+^ ions *in vitro* was further tested, and results showed that the pH of PBS increased slowly from 6.5 to 7.35 during the reaction of HSA-MnO_2_-CQ NPs with H^+^ ions (Figure S2A). Subsequently, the reaction kinetics of MnO_2_ with H_2_O_2_/H^+^ was investigated by testing the degradation behavior of MnO_2_ in HSA-MnO_2_-CQ NPs in different conditions, and the retained MnO_2_ was calculated by the decrease in its characteristic absorbance band. The results showed that MnO_2_ in HSA-MnO_2_-CQ NPs was stable at a pH of 7.4, but it degraded in the condition with a pH of 6.5 within 12 h. However, the retained MnO_2_ was rapidly decreased in the presence of H_2_O_2_ at a pH of 6.5 and was almost completely degraded within 3 h (Figure S2B). HSA-MnO_2_-CQ NPs reacted with H_2_O_2_/H^+^, resulting in the generation of paramagnetic Mn^2+^, and released Mn^2+^ at different pH values and at different concentrations of H_2_O_2_ measured by inductively coupled plasma atomic emission spectroscopy (Thermo). It was found that Mn^2+^ released rapidly from the NPs in the presence of H_2_O_2_ at a pH of 6.5 and was almost completely released from the NPs within 2 h when the H_2_O_2_ concentration was sufficient (2 mM). In contrast, bare or few Mn^2+^ were released from the NPs at a pH of 7.4 (Figure S2C). These results indicated that the HSA-MnO_2_-CQ NPs could relieve hypoxia and neutralize hydrogen ions to potentially regulate the bladder cancer microenvironment.

Once HSA-MnO_2_-CQ NPs reacted with H_2_O_2_, the nanoparticles were decomposed and gradually released the encapsulated chloroquine. Then, we evaluated the release of CQ from the nanoparticles with or without H_2_O_2_. As shown in Figure 1I, there was negligible CQ release in the PBS at a pH of 7.4, which indicated stabilization of HSA-MnO_2_-CQ NPs in the neutral environment. Once the nanoparticles decomposed in the acidic environment (pH 6.5), ~40% of CQ could be gradually released from HSA-MnO_2_-CQ NPs within 24 h. When incubated with H_2_O_2_, the released CQ was significantly increased to ~80% within 24 h.

### Restore the autophagy-inhibiting activity of chloroquine

Previous reports have shown that acidic extracellular pH hindered the autophagy-inhibiting ability of chloroquine ^12^, mainly due to the decreased intracellular uptake in the acidic microenvironment^ 11^. Our results confirmed this phenomenon in T24 bladder cancer cells. As shown in Figure S3B, there was significantly decreased cellular uptake of CQ in the acidic condition (pH 6.5) compared with the neutral condition (pH 7.4). In contrast, the cellular uptake of CQ in the acidic condition was increased 6.8-fold when CQ was encapsulated in HSA-MnO_2_-CQ NPs. Moreover, the cellular uptake of NPs in different conditions was evaluated using a Laser Scanning Confocal Microscope. The HSA-MnO_2_-CQ NPs were labeled with Cy7 (Cy7-NHS was conjugated with NH_2_ of HSA), and the cell uptake of the NPs by T24 cells in different conditions were observed. The results showed that the NPs could be effectively endocytosed by T24 cells in the neutral condition (pH 7.4), the acidic condition (pH 6.5) or the hypoxic condition, which were mainly located within the lysosomes (Figure S3A).

**Figure 2:**
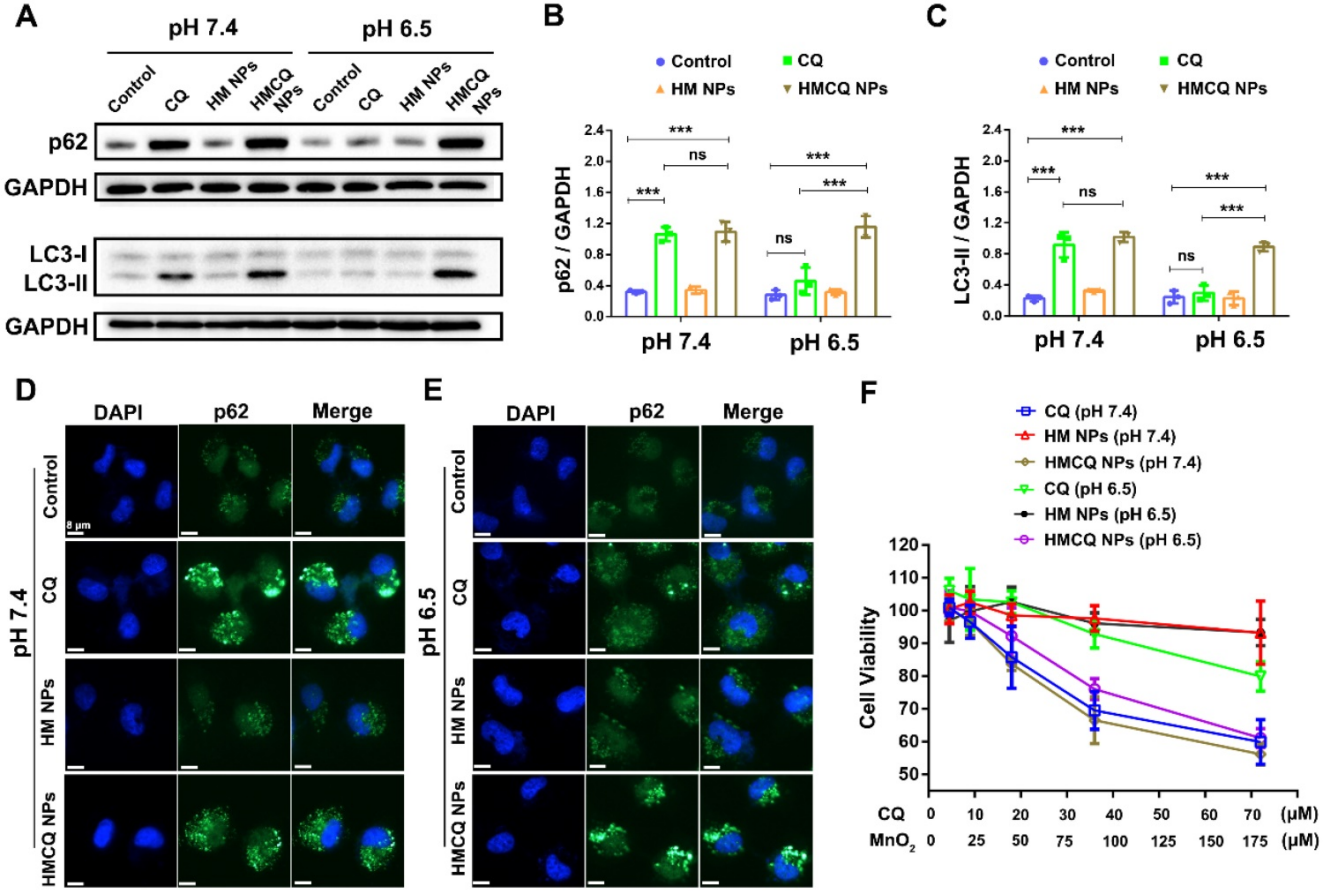
Autophagy-inhibiting activity of HSA-MnO_2_-CQ NPs. (A) WB analysis of p62 and LC3-II accumulation in T24 cells treated with CQ, HSA-MnO_2_ NPs and HSA-MnO_2_-CQ NPs at a pH of 7.4 or a pH of 6.5. (B, C) Quantification of LC3-II and p62 accumulation in T24 cells detected by WB. (D, E) p62 expression in T24 cells treated with CQ, HSA-MnO_2_ NPs and HSA-MnO_2_-CQ NPs at a pH of 7.4 or a pH of 6.5 by immunofluorescence staining. (F) Cytotoxic activity of CQ, HSA-MnO_2_ NPs and HSA-MnO_2_-CQ NPs at a pH of 7.4 or a pH of 6.5. Data are shown as the mean ± SD. *p<0.05; **p<0.01; ***p<0.001.

Then, we evaluated the ability of chloroquine or HSA-MnO_2_-CQ NPs to inhibit autophagy. The mechanism of autophagy inhibition by chloroquine closely relied on inhibiting the fusion of lysosomes with autophagosomes 32. Therefore, the amount of LC3-II and p62 delivered from autophagosomes can be used to estimate autophagic flux, and the accumulation of LC3-II and p62 in T24 cells was detected by Western blot (WB). The results showed that CQ induced accumulation of LC3-II and p62 only at the physiological pH of 7.4 but not in the acidic condition (pH 7.4, Figure 2A). Quantification of LC3-II and p62 accumulation also showed that CQ did not alter LC3-II and p62 levels at an acidic pH. However, HSA-MnO_2_-CQ NPs induced massive accumulation of LC3-II and p62 at both a pH of 7.4 and a pH of 6.5, indicating that HSA-MnO_2_-CQ NPs restored the autophagy-inhibiting ability of chloroquine in acidic conditions *via* increased intracellular uptake of chloroquine (Figure 2B and 2C, Figure S4). A similar phenomenon was also observed by immunofluorescent labeling, in which p62 accumulated within T24 cells (Figure 2D and 2E, Figure S5).

To further detect their influence on cell viability, we treated T24 bladder cancer cells with CQ, HSA-MnO_2_ NPs, or HSA-MnO_2_-CQ NPs at a pH of 7.4 and a pH of 6.5. The results showed increased cytotoxicity of CQ to T24 cells incubated at a pH of 7.4. However, HSA-MnO_2_-CQ NPs induced obvious cytotoxicity in T24 bladder cancer cells both at a pH of 7.4 and a pH of 6.5 (Figure 2F). These results showed that encapsulation of CQ into HSA-MnO_2_-CQ NPs could effectively restore autophagy-inhibiting activity, even in an acidic microenvironment.

### Enhanced autophagy inhibition and radiation therapy by relieving hypoxia

**Figure 3:**
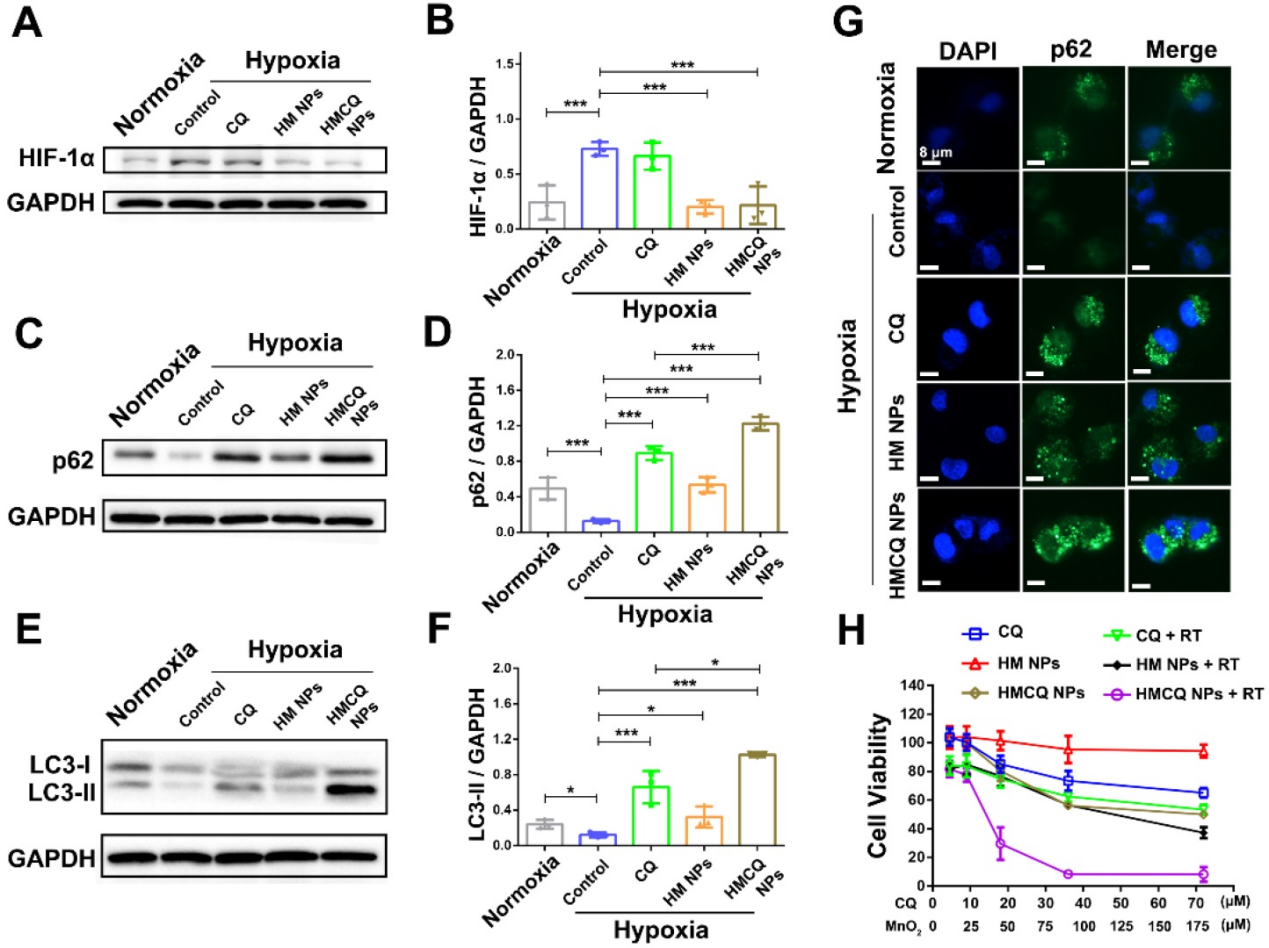
HSA-MnO_2_-CQ NPs enhanced autophagy inhibition and radiation therapy by ameliorating hypoxia. (A, B) HIF-1α protein in T24 bladder cancer cells after various treatments. (C, D) p62 levels in T24 bladder cancer cells after various treatments. (E, F) LC3-II levels in T24 bladder cancer cells after various treatments. (G) p62 expression in T24 cells after various treatments by immunofluorescence staining. (H) Cytotoxicity of CQ, HSA-MnO_2_ NPs and HSA-MnO_2_-CQ NPs at various concentrations with or without radiation under hypoxic conditions. Data are shown as the mean ± SD. *p<0.05; **p<0.01; ***p<0.001.

Hypoxia is an important hallmark of bladder cancers, and it promoted tumor growth and metastasis through various mechanisms 22-24. Hypoxia-induced autophagy also played an important role in promoting cancer cell growth via hypoxia-driven metabolic changes 33,34. We first investigated the autophagy-inhibiting ability of HSA-MnO_2_-CQ NPs in T24 bladder cancer cells in the hypoxic condition. As shown in Figure 3A and 3B, hypoxia-inducible-factor-1alpha (HIF-1α) levels were significantly upregulated in T24 bladder cancer cells when incubated in the hypoxic chamber. The effect of HSA-MnO_2_-CQ NPs on producing oxygen was also evaluated in T24 cells under hypoxic conditions using an oxygen probe. The results showed that the H_2_O_2_ concentration in cell medium released by T24 cells was 241.6 ± 49.5 μM, and significant amounts of oxygen were generated by NPs reacting with H_2_O_2_ released by the cancer cells, thus producing oxygen in situ (Figure S3C). After oxygen-supply treatment with HSA-MnO_2_ NPs or HSA-MnO_2_-CQ NPs, HIF-1α in T24 cells significantly decreased, and could be transported into the nucleus and lead to HIF target gene transcription 35. The accumulation of LC3-II and p62 in T24 cells decreased when incubated in hypoxic conditions, indicating that the hypoxic microenvironment potentially activated autophagy. The activation of autophagy by hypoxia could be inhibited when treated with an autophagy inhibitor, CQ, and HSA-MnO_2_-CQ NPs. As expected, HSA-MnO_2_-CQ NPs markedly blocked hypoxia-induced autophagic flux *via* the combination of hypoxia relief and autophagy inhibitor treatment (Figure 3C-G and Figure S4).

We then examined the cytotoxicity of different concentrations of CQ, HSA-MnO_2_, and HSA-MnO_2_-CQ NPs on T24 bladder cancer cells in hypoxic conditions. As shown in Figure 3H, HSA-MnO_2_-CQ NPs reduced cell viability more obviously than free CQ, which also indicated their superiority in inhibiting hypoxia-driven autophagy. Because they improved the tumor hypoxic microenvironment, we evaluated the combined therapeutic effect of HSA-MnO_2_-CQ NPs with radiation therapy. The results showed that cellular viability in the HSA-MnO_2_-CQ NPs + RT group (8.16%) was significantly lower than that of HSA-MnO_2_ NPs + RT (37.18%) and CQ + RT groups (53.34%), which indicated the therapeutic potential of HSA-MnO_2_-CQ NPs against bladder cancers.

### Regulation of tumor hypoxia and tumor acidic microenvironments

First, pharmaceutical analysis was performed by measuring CQ content in blood. T24 tumor-bearing mice were intravenously injected with HSA-MnO_2_-CQ NPs or free CQ. Blood samples were collected at 0.1, 0.5, 1, 2, 3, 6, 12 and 24 h. The concentrations of chloroquine in plasma were analyzed by HPLC. The results are shown in Figure S6A and 6C, and the plasma half-time of chloroquine in the HSA-MnO_2_-CQ NPs-treated group was 4.44 times higher than the free CQ-treated group, and the AUC of the HSA-MnO_2_-CQ NPs-treated group was 3.67 times higher than the free CQ-treated group, indicating improved pharmacokinetic behavior of chloroquine with HSA-MnO_2_-CQ NPs versus free CQ. Then, we evaluated the biodistribution of HSA-MnO_2_-CQ NPs after intravenous injection, and the biodistribution of HSA-MnO_2_-CQ NPs was performed by measuring chloroquine content in tumor tissues and major normal organs. After intravenous injection, HSA-MnO_2_-CQ NPs in tumor tissues gradually increased from 3 h and peaked at 24 h. Similarly, HSA-MnO_2_-CQ NPs accumulated in the liver in the first 24 h but disappeared at 48 h (Figure S6B). The results showed that HSA-MnO_2_-CQ NPs effectively accumulated in tumor tissues, potentially indicating their therapeutic advantages.

The accumulation of HSA-MnO_2_-CQ NPs in bladder cancer was further observed by magnetic resonance imaging (MRI). Mn^2+^ is an excellent T1-shortening agent in MRI, and its T1-weighted signal intensity was positively correlated with manganese concentration 36. The T1-weighted MR images were examined after intravenous injection of HSA-MnO_2_-CQ NPs into mice at 0, 12, 24, and 48 h. As shown in Figure S7A and 7B, there was no obvious MR signal enhancement in the tumor regions compared with muscular tissue before i.v. injection. However, the tumor area signal intensity was significantly increased due to the release of Mn^2+^ from HSA-MnO_2_-CQ NPs 12 h and 24 h postinjection. These results indicated that HSA-MnO_2_-CQ NPs accumulated in bladder tumors and decomposed to release Mn^2+^.

**Figure 4:**
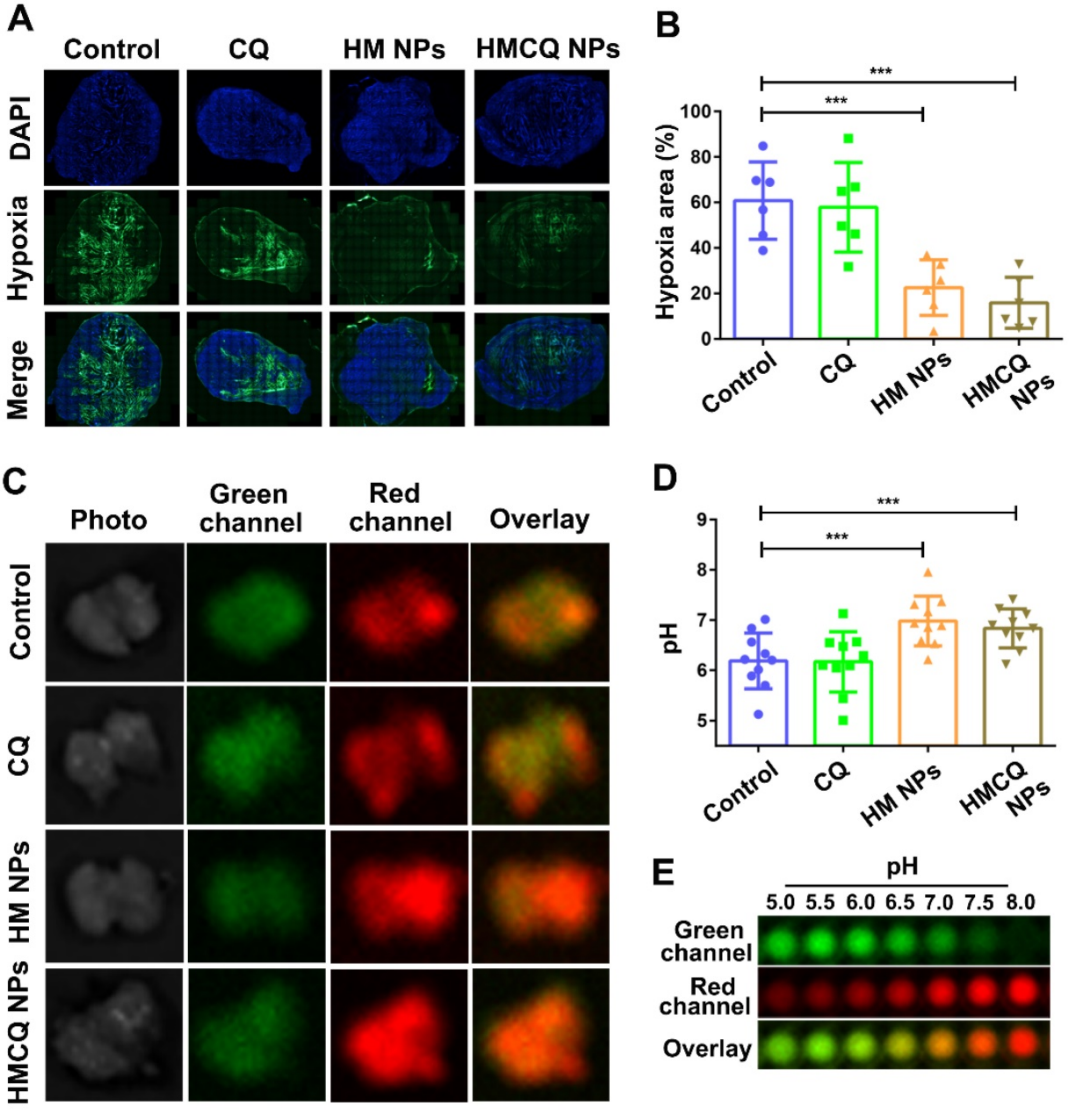
Regulation of tumor hypoxia and tumor pH by HSA-MnO_2_-CQ NPs. (A) Representative immunofluorescence images of whole tumor slices after hypoxia staining. Cell nuclei and hypoxic areas were stained with DAPI (blue) and anti-pimonidazole antibody (green), respectively. (B) Relative tumor hypoxia-positive areas determined from immunofluorescence images. (C) Multispectral fluorescence imaging (MSFI) of *ex vivo* tumor tissues after various treatments. (D) Tumor pH *ex vivo* after various treatments determined by MSFI. (E) The pH scale obtained using the dye in biological phantoms at various pH values. Data are shown as the mean ± SD. *p<0.05; **p<0.01; ***p<0.001.

Then, we investigated the effect of HSA-MnO_2_-CQ NPs for improving the hypoxic microenvironment of bladder cancer *in vivo*. A hypoxia-sensing probe (pimonidazole) was utilized to visualize the tumor hypoxic areas. After treatment with HSA-MnO_2_ and HSA-MnO_2_-CQ NPs, the hypoxia within tumor tissues was significantly relieved (Figure 4A, Figure S7C). Specifically, semiquantification data showed that the tumors in control groups were highly hypoxic (60.76% positive area), but the tumors treated with HSA-MnO_2_ NPs or HSA-MnO_2_-CQ NPs exhibited significantly decreased hypoxia areas (21.51% and 15.80%) (Figure 4B). The hypoxia relief could be attributed to the reaction of HSA-MnO_2_-CQ NPs and H^+^/H_2_O_2_ and sustained oxygen generation in bladder cancer tissues.

To measure intratumoral pH, a pH mapping probe (multispectral fluorescence imaging (MSFI) with a pH-sensitive fluorescent dye (SNARF-4F)) was used as previously reported 37. MSFI of tumor tissues was performed after treatment with PBS, CQ, HSA-MnO_2_ NPs, and HSA-MnO_2_-CQ NPs. MSFI images of tumors were correlated to local pH from the calibration curves obtained earlier with biological tissue-like phantoms (Figure 4E, Figure S7D and 7E). The results showed that the mean tumor pH was significantly increased after the treatment of HSA-MnO_2_-CQ NPs (pH=6.83) compared with treatment with PBS (pH=6.18), indicating the ability of HSA-MnO_2_-CQ NPs to regulate the tumor acidic microenvironment (Figure 4C, D).

### Detection of autophagy-inhibiting activity *in vivo*

**Figure 5:**
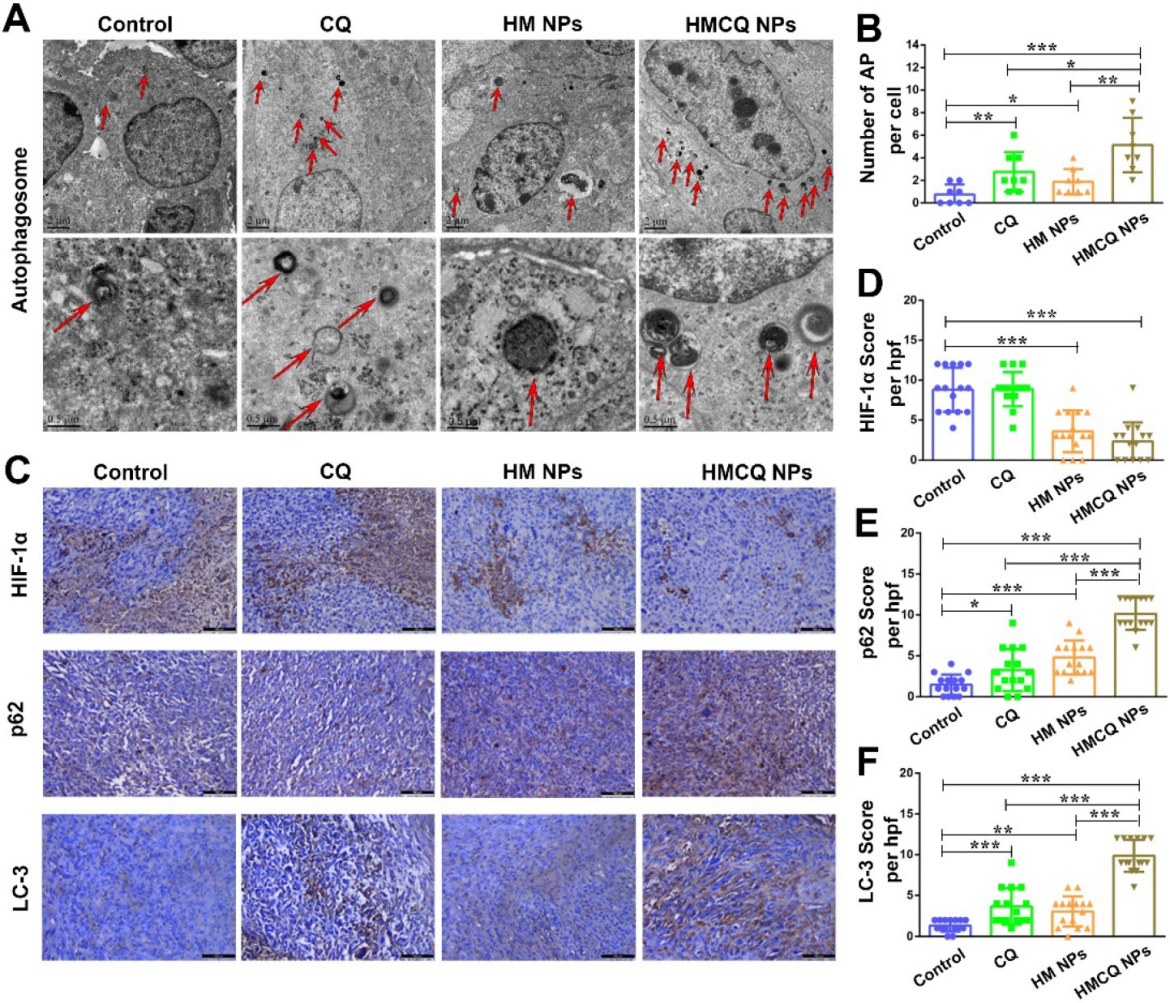
The autophagy-inhibiting activity of HSA-MnO_2_-CQ NPs* in vivo*. (A) Transmission electron microscopy micrographs of T24 tumors treated with CQ, HSA-MnO_2_ NPs and HSA-MnO_2_-CQ NPs. The red arrows indicate autophagosomes. (B) Quantification of autophagosomes (AP) in 8 random fields of TEM micrographs. (C) Immunohistochemical staining for HIF-1α, p62 and LC3 in T24 tumors treated with CQ, HSA-MnO_2_ NPs and HSA-MnO_2_-CQ NPs. (D, E, F) Quantification of HIF-1α expression, p62 expression and LC3 expression in immunohistochemical staining images. Data are shown as the mean ± SD. *p<0.05; **p<0.01; ***p<0.001.

Since the HSA-MnO_2_-CQ NPs could regulate the tumor in hypoxic and acidic microenvironments, we further investigated the autophagy-inhibiting ability of HSA-MnO_2_-CQ NPs* in vivo*. Autophagosomes are membrane‑bound compartments that contain cytoplasmic material and/or organelles 38. As shown in Figure 5A, only a few autophagosomes were observed in the control group (0.75 ± 0.88 autophagosomes per cell). The accumulation of autophagosomes was detected in tumors treated with HSA-MnO_2_-CQ NPs (5.12 ± 2.41) compared with CQ (2.75 ± 1.75) or HSA-MnO_2_ NPs (1.87 ± 1.12) (Figure 5B). The autophagic regulation proteins (p62 and LC3) were then estimated by immunohistochemistry (Figure 5C). HSA-MnO_2_-CQ NPs significantly decreased the expression of HIF-1α protein in T24 tumors (Figure 5D), upregulated the expression of p62 and LC3, and subsequently inhibited autophagic flux (Figure 5E, F). All of these results indicated that the HSA-MnO_2_-CQ NPs had remarkable autophagy inhibition efficacy on bladder tumors via synergistically providing hypoxia relief and autophagy inhibitor treatment.

### Enhanced therapeutic efficiency of HSA-MnO_2_-CQ NPs in T24-bearing mice

**Figure 6:**
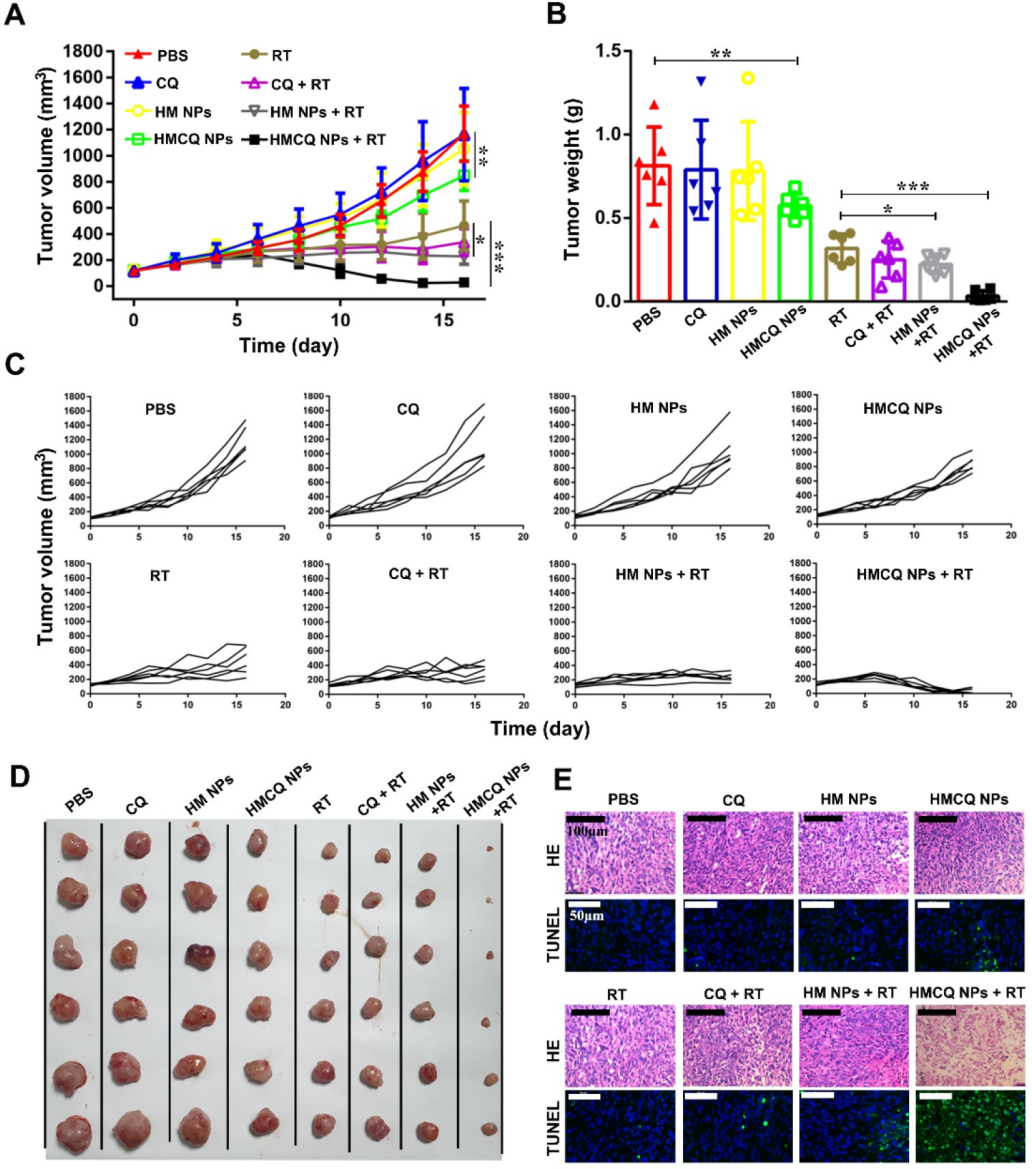
Enhanced efficiency of bladder cancer therapy* in vivo*. (A) Tumor growth curves after different treatments in the presence or absence of radiation. (B) Tumor weight collected on day 16. (C) Tumor growth curve of each mice after different treatments. (D) Photos of tumor masses collected on day 16. (E) H&E staining and TUNEL of tumor sections obtained from mice on day 16. Data are shown as the mean ± SD. *p<0.05; **p<0.01; ***p<0.001.

Previous reports showed TME remodeling is an effective strategy to improve cancer treatment 39-42. HSA-MnO_2_-CQ NPs were able to relieve tumor hypoxia, increase tumor pH, and inhibit autophagic flux, which was speculated to sensitize radiation therapy (RT) against bladder cancer. To test this hypothesis, we measured tumor growth in T24 xenograft mice treated with PBS, CQ, HSA-MnO_2_ NPs, and HSA-MnO_2_-CQ NPs with or without radiation therapy. Compared with the PBS control group, tumor growth profiles showed that free CQ or HSA-MnO_2_ NP treatment did not inhibit tumor growth. However, after HSA-MnO_2_-CQ NP treatment, 27.5% of inhibition in tumor growth was achieved. More importantly, 97.5% of inhibition in tumor growth was observed in the HSA-MnO_2_-CQ NPs + RT combination treatment, indicating the remarkable sensitization effect of HSA-MnO_2_-CQ NPs (Figure 6A-D). H&E and TUNEL staining of tumor sections on day 16 also showed that HSA-MnO_2_-CQ NPs + RT treatment induced significant tumor necrosis areas and cell nucleus dispersion (Figure 6E). The mice did not display obvious bodyweight changes with various treatments (Figure S9A).

The results demonstrated that the significant anti-tumor effect achieved by the combination therapy could be ascribed to enhanced autophagy inhibition and radiation sensitization. Previous reports also showed that radiation induced autophagy to promote tumor survival, which further led to tumor radiation resistance 27,28. Our IHC staining of tumor sections on day 6 also showed that both p62 (p62 score=1.06±1.16) and LC3 expressions (LC-3 score=0.73±0.70) slightly decreased in tumor tissues after radiation therapy compared with tumors treated with PBS (p62 score=1.53±1.07, LC-3 score=1.2±0.86). When the tumors were treated with HSA-MnO_2_-CQ NPs before radiation therapy, p62 and LC3 expressions were significantly enhanced, which demonstrated their ability to block autophagic flux (Figure S8).

### Detection of HSA-MnO_2_-CQ NPs safety *in vivo*

To further evaluate the safety of HSA-MnO_2_-Ce6 NPs *in vivo*, we tested blood samples and organs of healthy Balb/c nude mice injected with PBS or HSA-MnO_2_-CQ NPs. ALT and AST levels were tested to evaluate hepatic function, and UREA and CREA levels were used to evaluate renal function. There was no significant difference in hepatic and renal function between the treatment of PBS and HSA-MnO_2_-CQ NPs (Figure S9B). HE-stained sections of the organs (heart, liver, spleen, lung, and kidney) also showed no apparent lesions (no necrosis, edema, inflammatory infiltration or hyperplasia) after treatment with PBS or HSA-MnO_2_-CQ NPs (Figure S9C).

## Conclusion

In summary, by taking advantage of the strong affinity between negatively charged MnO_2_ and positively charged CQ, we fabricated HSA-MnO_2_-CQ NPs to regulate the tumor microenvironment. The NPs efficiently generated O_2_ and increased pH *in vitro* and* in vivo* after reaction with H^+^/H_2_O_2_ and then released the encapsulated CQ in a H^+^/H_2_O_2_ concentration-dependent manner. HSA-MnO_2_-CQ NPs were able to restore the autophagy-inhibiting activity of chloroquine in the acidic microenvironment by increasing its intracellular uptake. In addition, the NPs also markedly blocked hypoxia-induced autophagy by relieving hypoxia. *In vivo* studies showed that HSA-MnO_2_-CQ NPs synergistically regulated the tumor microenvironment and inhibit autophagic flux and then subsequently sensitized radiation therapy to achieve excellent therapeutic outcomes.

## Methods and Materials

### Materials

HSA was obtained from CSL Behring AG (Switzerland). CQ was obtained from J&K Scientific Ltd. (China). Potassium permanganate (KMnO_4_) and hydrogen peroxide (H_2_O_2_, 30 wt%) were obtained from Sino pharm Chemical Reagent Co. (China). 4',6-diamidino-2-phenylindole dihydrochloride (DAPI) was obtained from Molecular Probes (USA). RPMI-1640 medium was obtained from Gibco (Grand Island, NY, USA). The LC-3B antibody, p62 antibody, and HIF-1α antibody were obtained from Abcam (UK). The pH-sensitive fluorescent dye SNARF-4F was obtained from Life technologies (USA). The hypoxia probe pimonidazole (Hypoxyprobe-1 plus kit) was obtained from Hypoxyprobe Inc. (USA), and 1×Phosphate buffer solution (PBS) and deionized water were used in the experiments. All male Babl/c nude mice (17~20 g) were obtained from Yangzhou University Medical Center.

### Preparation and Characterization of HSA-MnO_2_-CQ NPs

The HSA-MnO_2_-CQ NPs were obtained *via* a two-step deposition method according to our previous report 31. Briefly, 25 mg of KMnO_4_ was dissolved in deionized water and 30 mg of chloroquine was dissolved in 2 mL deionized water. In the first cycle, 2 mL of KMnO_4_ was dropped into 50 mg/mL HSA (2 mL) under vigorous stirring at 37 °C to form the basal HSA-MnO_2_ NPs. Five minutes later, 200 µL of chloroquine was added in a dropwise manner into the HSA-MnO_2_ NPs. Then, the KMnO_4_ and chloroquine solutions were added alternately for five cycles to form primal HSA-MnO_2_-CQ NPs. Free chloroquine was removed by an ultrafiltration device (Millipore 8400, ultrafiltration membrane MW:30 KD). Finally, an extra KMnO_4_ solution was added to form the external shelter to obtain the final HSA-MnO_2_-CQ NPs. They were further purified by removing free small molecules with an ultrafiltration membrane. Then, the solutions were filtered through a filter membrane (pore size 0.22 μm). The NP dispersions were stored in 4 °C for further use. Concentrations of MnO_2_ were measured using inductively coupled plasma atomic emission spectroscopy (Thermo), and the concentrations of chloroquine were measured with a UV-vis Spectrometer (UV2450, Shimadzu Corp.). The molar ratio of CQ to MnO_2_ in the nanoparticles was 1:2.5.

The morphology and particle size of NPs were measured by transmission electron microscopy (TEM) (Hitachi H-7650). The dynamic light scattering (DLS) and zeta potential of the NPs were measured using a Brookhaven apparatus (Brookhaven Instruments, USA). The colloidal stability of HSA-MnO_2_-CQ NPs was investigated in PBS and serum at 37 °C or 25 °C by measuring their mean diameter with DLS. The concentration of oxygen generated by the reaction of HSA-MnO_2_-CQ NPs with H_2_O_2_ at different pH was measured in a sealed chamber coupled with an oxygen electrode (Dissolved Oxygen Meter, AZ-8402, Guangdong, China). The pH change in the reaction of HSA-MnO_2_-CQ NPs and H_2_O_2_ in PBS buffer was measured by Oakton pH 1100 (Termo Fisher Scientific Inc., USA).

Release of chloroquine from HSA-MnO_2_-CQ NPs at different pH values (pH 7.4 and 6.5) with or without H_2_O_2_ was investigated. HSA-MnO_2_-CQ NPs were suspended in 10 mL of PBS (pH 7.4 and 6.5) at 37 °C. H_2_O_2_ was added at 0, 3, 6, 12 and 18 h. The release system was maintained at 37 °C under shaking. The release medium was sampled with 1 mL each time, and UV/vis spectrophotometry was used to determine the percentage of released chloroquine. The sample was added back to the original release system. The amount of chloroquine loaded in HSA-MnO_2_-CQ NPs was determined by the UV-Vis absorbance at 334 nm. The percentages of release of chloroquine were calculated according to a formula, Release percentage (%) = M_r_/M_t_, where M_r_ is the amount of released chloroquine, and M_t_ is the total amount of loaded chloroquine.

### Autophagy-inhibiting activity of HSA-MnO_2_-CQ NPs in acidic conditions

To investigate the autophagy-inhibiting activity of HSA-MnO_2_-CQ NPs in acidic condition *in vitro*, T24 cells were plated in standard RPMI-1640 buffered at pH 7.4. The next day, the medium was replaced with media buffered at different pH (7.4 and 6.5). After 24 h of exposure to medium at different pH, cells were treated with PBS, CQ (36 μM), HSA-MnO_2_ NPs (90 μM) and HSA-MnO_2_-CQ NPs (CQ 36 μM, MnO_2_ 90 μM) for 12 h and then collected. The expression of LC-3 and p62 in cells were detected by Western blot. Briefly, the cells were lysed in RIPA buffer. Samples were separated by SDS-PAGE and transferred to PVDF membranes (Millipore, France). After blocking with 5% skim milk at room temperature for 1 h, the membranes were incubated with the primary antibodies rabbit anti-p62 (1:5000) and rabbit anti-LC3 (1:1000) at 4 °C overnight. The membranes were washed with TBST buffer and incubated with HRP-conjugated secondary antibodies, such as anti-mouse-IgG/anti rabbit-IgG for 2 h at 37 °C. Immunoblotted proteins were visualized by an imaging system (Bio-Rad, CA, USA). Quantification of LC3-II and p62 were analyzed by ImageJ software. For further immunofluorescence staining analysis, T24 cells were seeded in 6-well plates. The treatments were the same as for the above experiments. The p62 expression in cells was detected by immunofluorescence staining. Briefly, the cells were fixed with 4% paraformaldehyde, permeabilized with 0.1% Triton X-100 at 37 °C for 15 min and stained with rabbit anti-p62 antibody (1:200) at 4 °C overnight. After washing in PBS, a secondary antibody (1:100) conjugated to FITC was added and incubated for 2 h at 37 °C. Then, cells were incubated with DAPI. The images were examined under a fluorescence microscope.

For the MTT assay under acidic and neutral condition, T24 cells were seeded onto 96-well plates in standard RPMI-1640 buffered at a pH of 7.4. The next day, the medium was replaced with media buffered at different pH (7.4 and 6.5). After 24 h of exposure to medium at different pH, cells were treated with PBS, CQ, HSA-MnO_2_ NPs and HSA-MnO_2_-CQ NPs for 12 h. Medium was replaced by fresh medium, and 24 h later, 100 µL MTT solution was then added into each well. After 3 h, 100 µL of DMSO was introduced into each well. The absorbance at 570 nm was detected.

### Autophagy-inhibiting activity of HSA-MnO_2_-CQ NPs in hypoxic conditions

To investigate the autophagy-inhibiting activity of HSA-MnO_2_-CQ NPs in hypoxic conditions *in vitro*, T24 cells were seeded onto plates in standard RPMI-1640 buffered at pH 7.4. For the hypoxia groups, plates were covered with paraffin for 6 h to provide a hypoxic environment. Then, T24 cells were incubated with different concentrations of CQ (36 μM), HSA-MnO_2_ NPs (90 μM) and HSA-MnO_2_-CQ NPs (CQ 36 μM, MnO_2_ 90 μM) for 12 h. Cells were collected, and the expression of HIF-1α, LC-3 and p62 in cells were detected by Western blot. The WB data were quantified using ImageJ software. For further immunofluorescence staining assays, T24 cells were seeded in 6-well plates. The treatment was the same as the above experiments. p62 expression in cells were detected by immunofluorescence staining.

To study the MTT assay under hypoxic conditions with or without radiation, T24 cells were seeded onto 96-well plates in standard RPMI-1640. Plates were covered with paraffin for 6 h to provide a hypoxic environment. Then, T24 cells were incubated with different concentrations of CQ, HSA-MnO_2_ NPs and HSA-MnO_2_-CQ NPs for 12 h. Then, the radiation efficiency was 3 Gy/min, and radiation lasted for 2 min. After radiation (0 or 6 Gy), different treatments were aborted, and cells were cultured for another 24 h. Then, the MTT assay was performed.

### Regulation of tumor hypoxia and tumor acidic pH by HSA-MnO_2_-CQ NPs

All the animals were obtained from Yangzhou University Medical Center (Yangzhou, China) and received care in accordance with Institution Animal Care and Use Committee (IACUC) of Nanjing University. T24 tumors were developed by subcutaneously implanting 5×10^6^ T24 cell suspensions in the lower backs of the mice. When the tumors were approximately 150 mm^3^ (tumor volume=a*(b/2)^2^, a=major axis, b=minor axis), mice were intravenously injected with PBS, CQ (40 μmol/kg), HSA-MnO_2_ NPs (100 μmol/kg) and HSA-MnO_2_-CQ NPs (CQ 40 μmol/kg, MnO_2_ 100 μmol/kg) once per day for 4 days.

To observe the tumor hypoxia area, a hypoxyprobe (pimonidazole) was used to observe pimonidazole-stained tumor hypoxia areas visually 24 h after treatment. Tumors were surgically excised 90 min after intraperitoneal injection with pimonidazole hydrochloride (60 mg/kg). Frozen sections of tumors were collected. The tumor sections were incubated with mouse anti-pimonidazole antibody (dilution 1:100, Hypoxyprobe Inc.) and Alexa 488-conjugated goat anti-mouse secondary antibody (dilution 1:200, Jack-son Inc.) as instructed. Nuclei were stained with DAPI.

To measure the tumor pH 24 h after treatments, a pH-sensitive fluorophore SNARF-4F was used for *ex vivo* tumor pH imaging following an established protocol 37. Mice were intravenously injected with dye (1 nmol of SNARF-4F in 200 μL of PBS). Animals were sacrificed 20 min following injections, and tumor tissue was immediately harvested, cut in half and imaged with *in vivo* imaging system (IVIS Lumina, USA). The tumor pH was calculated according to published protocols.

### The autophagy-inhibiting activity of HSA-MnO_2_-CQ NPs* in vivo*

To investigate the autophagy-inhibiting activity of HSA-MnO_2_-CQ NPs* in vivo*, T24 tumor bearing mice (tumor volume ~150 mm^3^) were intravenously injected with PBS, CQ (40 μmol/kg), HSA-MnO_2_ NPs (MnO_2_ 100 μmol/kg) and HSA-MnO_2_-CQ NPs (CQ 40 μmol/kg, MnO_2_ 100 μmol/kg) once per day for 4 days. The tumors were collected 24 h after treatment. For transmission electron microscopy observations, tumors were fixed overnight in ice-cold glutaraldehyde (3% in 0.1 M cacodylate buffer, pH=7.4) before being postfixed in 1% OsO_4_ for 45 min. Then, the tissues were dehydrated in a graded series of 70% to 100% acetone and embedded in Epon812. Subsequently, ultrathin sections were stained by uranium tetraacetate and lead citrate trihydrate and observed under a transmission electron microscope (JEM-1200EX, JEOL, Japan). For immunohistochemistry assay, tumor tissues were fixed in 10% formalin, paraffinized, and cut into 5 μm-thick sections. The slides were incubated with HIF-1α, LC3 and p62 antibodies and then incubated with HRP-conjugated antibodies. The slides were visualized with a DAB Horseradish Peroxidase Color Development Kit and counterstained with hematoxylin. The images were analyzed by ImageJ software.

### The therapeutic efficiency of HSA-MnO_2_-CQ NPs in T24-bearing mice

T24 tumor bearing mice (150 mm^3^) were divided into eight groups (6 mice in each group): PBS, CQ, HSA-MnO_2_ NPs, HSA-MnO_2_-CQ NPs, radiation therapy (RT), CQ with RT, HSA-MnO_2_ NPs with RT and HSA-MnO_2_-CQ NPs with RT (6 Gy). Mice were injected with different drugs *via* the tail vein (CQ 40 μmol/kg, MnO_2_ 100 μmol/kg) once per day for 4 days. The time at first injection was designated day 0. X-ray radiation therapy was performed at day 4. The tumor size and body weight were recorded. On day 16, mice were sacrificed, and tumors were collected. Photographs were taken by a high-quality camera, and hematoxylin and eosin (HE) staining and TUNEL assays of the tumors were performed.

### Statistical analysis

Statistical analysis involved two-sided Student t-test for two groups and one-way ANOVA for multiple groups. P < 0.05 was considered statistically significant.

## Supplementary Material

Supplementary figures.

## Abbreviations

ROS: reactive oxygen species
TME: tumor microenvironment
HSA: human serum albumin
MnO_2_: manganese dioxide
CQ: chloroquine
HMCQ NPs: HSA-MnO_2_-CQ NPs
HM NPs: HSA-MnO_2_ NPs
KMnO_4_: Potassium permanganate
H_2_O_2_: hydrogen peroxide
