# Supplementary material for: Synergy of Tumor Microenvironment Remodeling and Autophagy Inhibition to Sensitize Radiation for Bladder Cancer Treatment
Source: Theranostics. 2020 Jun 19;10(17):7683–96. doi: 10.7150/thno.45358 (PMC7359086; doi:10.7150/thno.45358)
Supplement: Supplementary file 1 — Supplementary figures. [file thnov10p7683s1.pdf]

Synergy of tumor microenvironment remodeling and autophagy inhibition to sensitize radiation for bladder cancer treatment

Tingsheng Lin[1,†], Qing Zhang[1,†], Ahu Yuan[2], Baojun Wang[1], Feifei Zhang[1], Yuanzhen Ding[1], Wenmin Cao[1], Wei Chen[1], Hongqian Guo[1,*].

Affiliations:
[1]Department of Urology, Drum Tower Hospital, Medical school of Nanjing University, Institute of Urology, Nanjing University, Nanjing, Jiangsu, 210008, China.
[2]State Key Laboratory of Pharmaceutical Biotechnology, Medical School of Nanjing University, Nanjing 210093, Jiangsu, China.

[†]These authors contributed equally to the article

[*]Author for correspondence:
Hongqian Guo, Ph. D.
Address: Department of Urology, Drum Tower Hospital, Medical school of Nanjing University, Institute of Urology, Nanjing University, Nanjing 210008, Jiangsu, China
E-mail: dr.ghq@nju.edu.cn

Figures

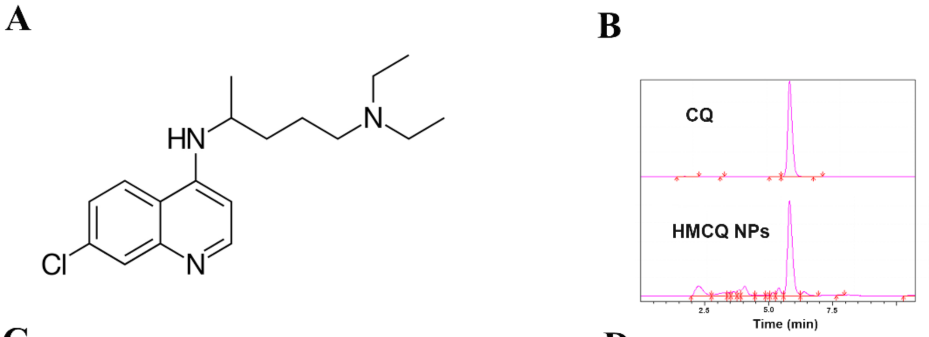

C

	Particle Size (nm)	Zeta Potential (mV)	Encapsulation Efficiency (%)
HM NPs	8.19 ± 3.22	-27.94 ± 2.73	0
HMCQ NPs (1 cycle)	15.25 ± 2.69	-24.13 ± 2.49	25.75 ± 3.38
HMCQ NPs (2 cycle)	19.27 ± 3.67	-19.39 ± 3.58	32.10 ± 4.17
HMCQ NPs (3 cycle)	31.92 ± 6.16	-15.84 ± 3.63	34.56 ± 5.99
HMCQ NPs (4 cycle)	38.01 ± 5.17	-12.34 ± 4.14	36.88± 3.66
HMCQ NPs (5 cycle)	44.81 ± 4.43	-6.37 ± 3.78	42.04 ± 4.71
HMCQ NPs (Final)	55.35 ± 5.06	-12.80 ± 3.51	39.19 ± 4.55

Encapsulation Efficiency (%) =Encapsulated Drug / Total Drug added *100%

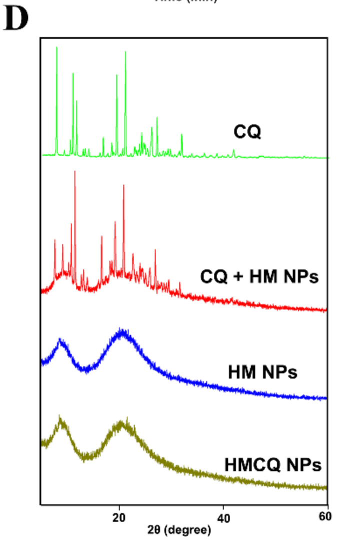

Figure S1. (A) The chemical structure of chloroquine. (B) The HPLC profile of chloroquine extracted from HSA-MnO_2-CQ NPs. (C) Characterization of HSA-MnO_2-CQ NPs in the growth process. (D) The XRD patterns of CQ, HM NPs + CQ physical mixture, HM NPs and HMCQ NPs.

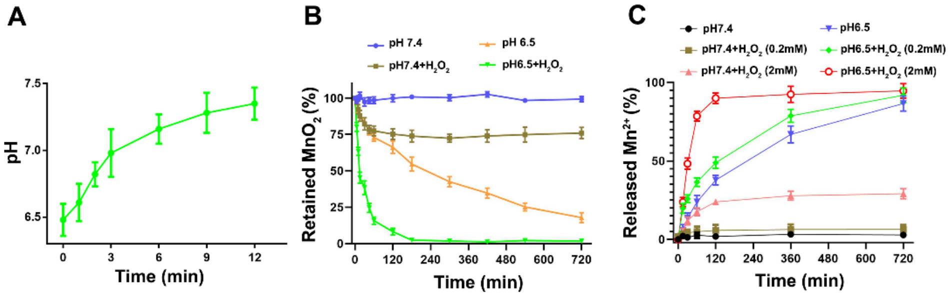

Figure S2. (A) The ability of HSA-MnO_2-CQ NPs to consume H^+ ions, results showed the pH of PBS increased slowly from 6.5 to 7.35 during the reaction of HSA-MnO2-CQ NPs with H+ ions. (B) The reaction kinetics of MnO_2 with H_2O_2/H^+. (C) The release profile of Mn^{2+} from the HSA-MnO_2-CQ NPs in different conditions.

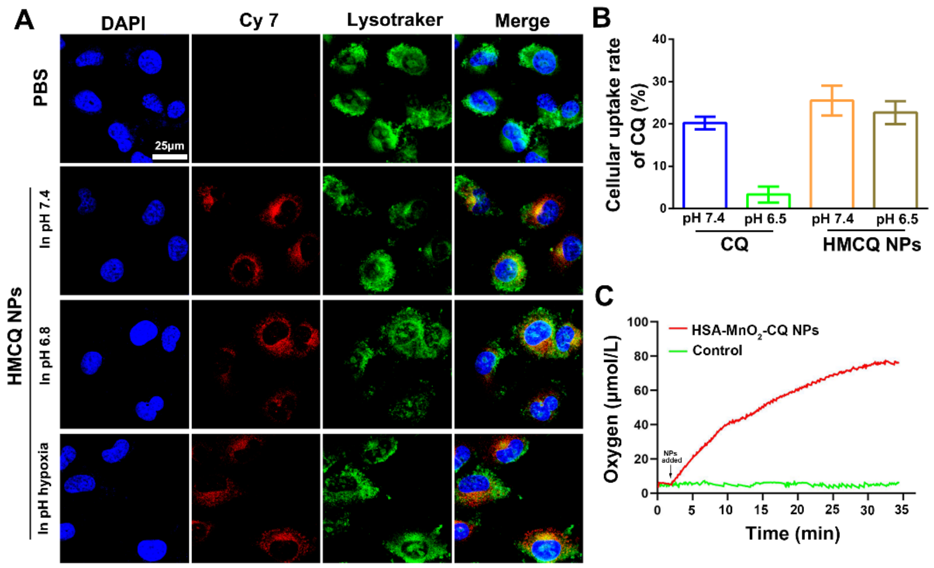

Figure S3. (A) The cellular uptake of Cy7 labelled NPs by T24 cells in different conditions. (B) T24 bladder cancers were treated with CQ or HSA-MnO_2-CQ NPs in acidic condition (pH 6.5) and neutral condition (pH 7.4). Cellular uptake of chloroquine was determined by HPLC. (C) O_2 assay in living cells in vitro.

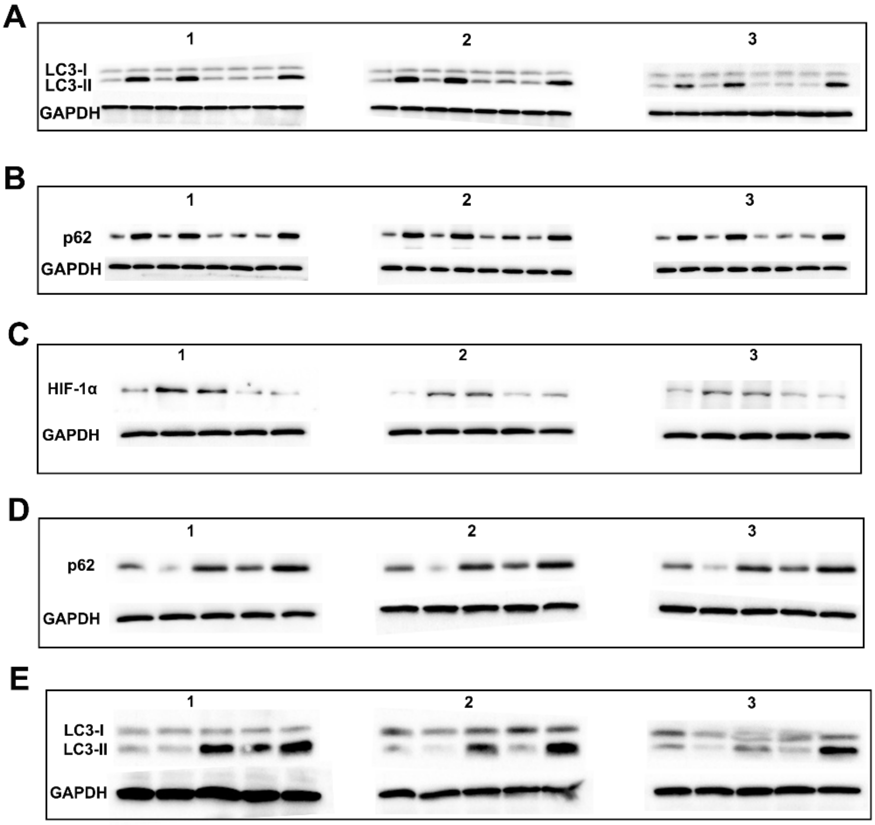

Figure S4. (A,B) WB examinations of p62 and LC3-II accumulation in T24 cells treated with CQ, HSA-MnO_2 NPs and HSA-MnO_2-CQ NPs at pH 7.4 or pH 6.5. (C,D,E) WB examinations of HIF-1α, p62 and LC3-II accumulation in T24 bladder cancer cells after various treatments in hypoxia condition.

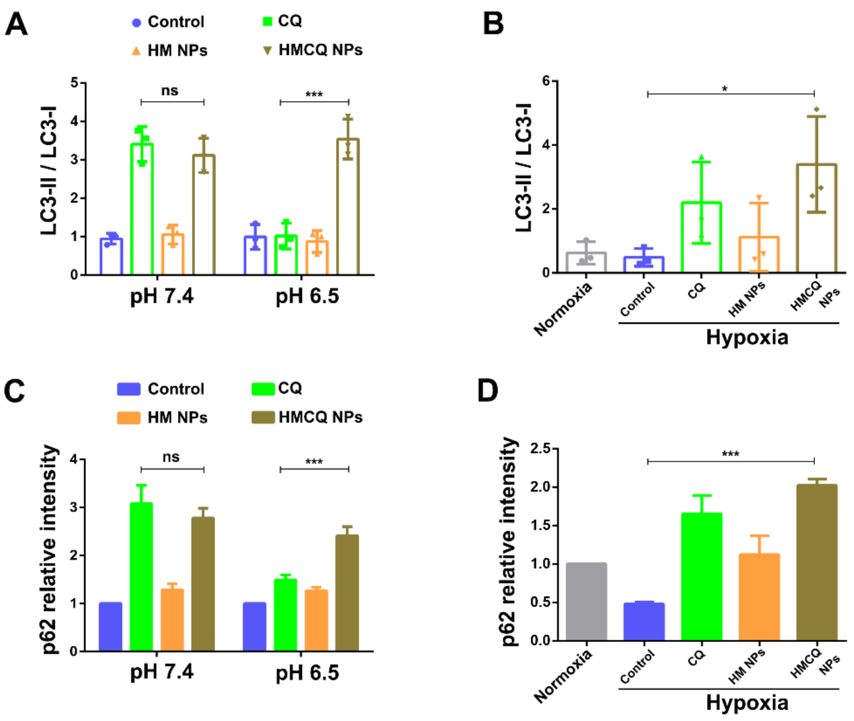

Figure S5. (A) The quantification of LC3-II/I in Figure S4A. (B) The quantification of LC3-II/I in Figure S4E. (C) The quantification of p62 in Figure 2D and 2E. (D) The quantification of p62 in Figure 3G.

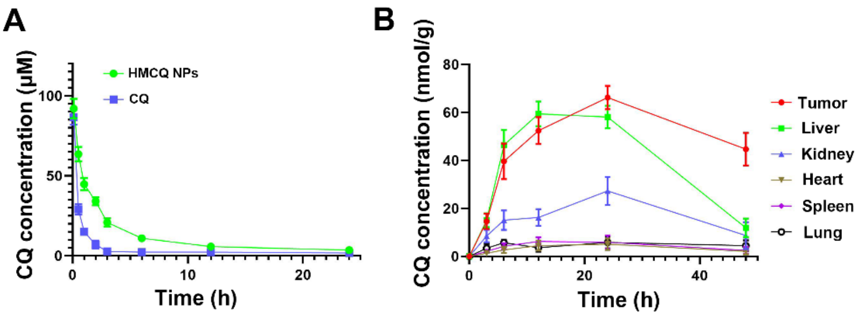

C

Parameter	Unit	CQ	HMCQ NPs
t1/2	h	0.29±0.04	1.290±0.09
Cmax	nmol/ml	106.23±9.82	89.504±5.52
AUC (0-t)	nmol/ml*h	45.56±3.35	167.120±16.01
AUMC	nmol/ml*h*h	19.61±4.16	312.096±47.7

Figure S6. (A) The blood circulation of HSA-MnO_2-CQ NPs or free CQ in vivo, blood samples were collected and analyzed with HPLC at different time points. (B) The tumor accumulation and biodistribution of HSA-MnO_2-CQ NPs after intravenous injection. (C) Pharmaceutical analysis of HSA-MnO_2-CQ NPs or free CQ after intravenous injection.

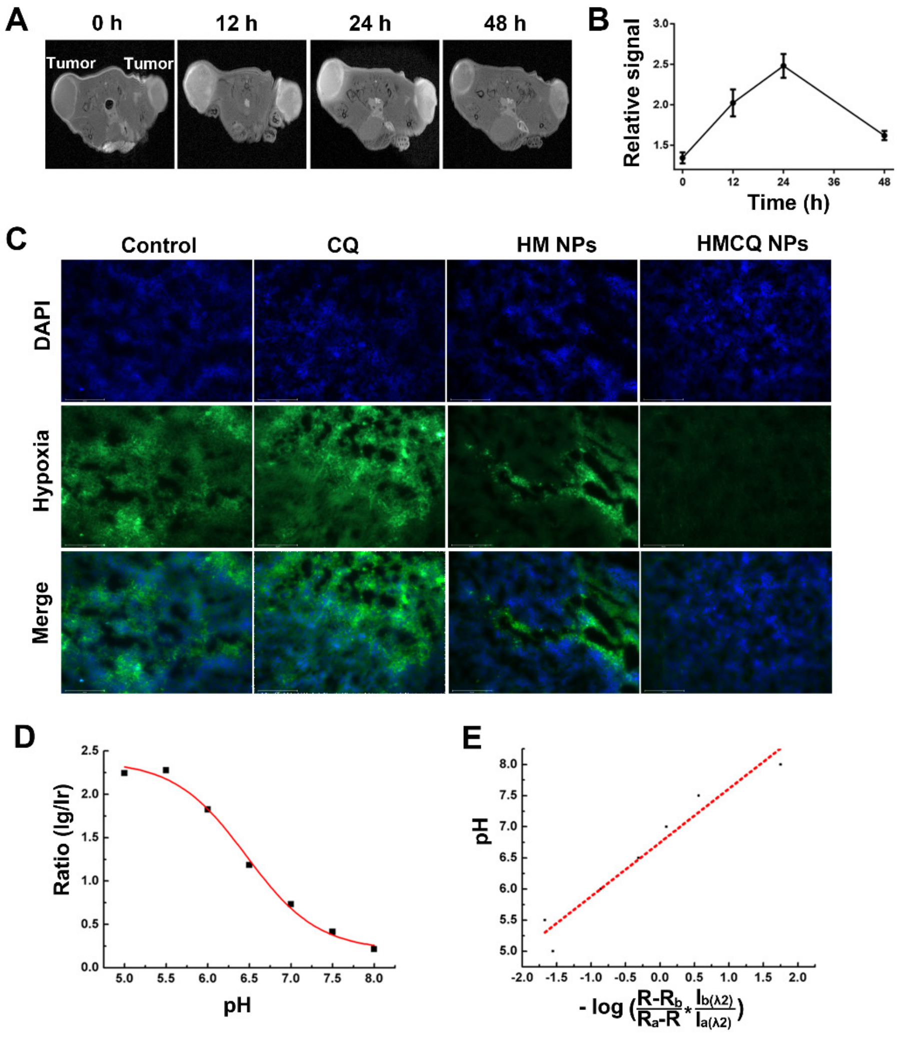

Figure S7. (A) The accumulation of HSA-MnO_2-CQ NPs in mice bearing T24 tumor was observed by MRI. The T1-weighted MR images were examined after intravenous injection of HSA-MnO_2-CQ NPs into mice at 0, 12, 24, and 48 h. (B) Relative signal intensity (RSI) was calculated from the ratio between signals in tumor areas and normal tissue areas. (C) Representative immunofluorescence images of tumor slices (high power field) after hypoxia staining. Cell nucleus and hypoxia areas were stained with DAPI (blue) and anti-pimonidazole antibody (green). (D) The ratios (R=Ig/Ir) versus pH graph, the ratio R was calculated from the values Ig and Ir which were obtained from fluorescent image in Figure 4e using image-J program. The Boltzmann fit of data points R using Eq.1 【$R_{pH}=(R_a-R_b)/[1+\exp(pH-pH_{inf})/\Delta pH]+R_b$】, the values for fit parameter were Ra= 2.387, Rb=0.197, pH_{inf} = 6.461, and ΔpH = 0.43. (E) Shows graph of - log(...) term versus pH, the ratio R was obtained from the Boltzmann fit in (a) and Ia(λ2) and Ib(λ2) are fluorescence intensities at 640nm (red channel) obtained from image in (a) at pH 5.0 and pH 8.0 using imageJ program, $I_{a(\lambda2)}$= 46.826 and $I_{b(\lambda2)}$= 18.596. Finally, the intercept of linear fit of data points in (b) is pKa according to Eq. 2 【$pH=pKa^*-\log[(R_a-R_b)/(R_a-R)^* I_{b(\lambda2)}/ I_{a(\lambda2)}]$】. The

obtained pKa of SNARF was 6.746.

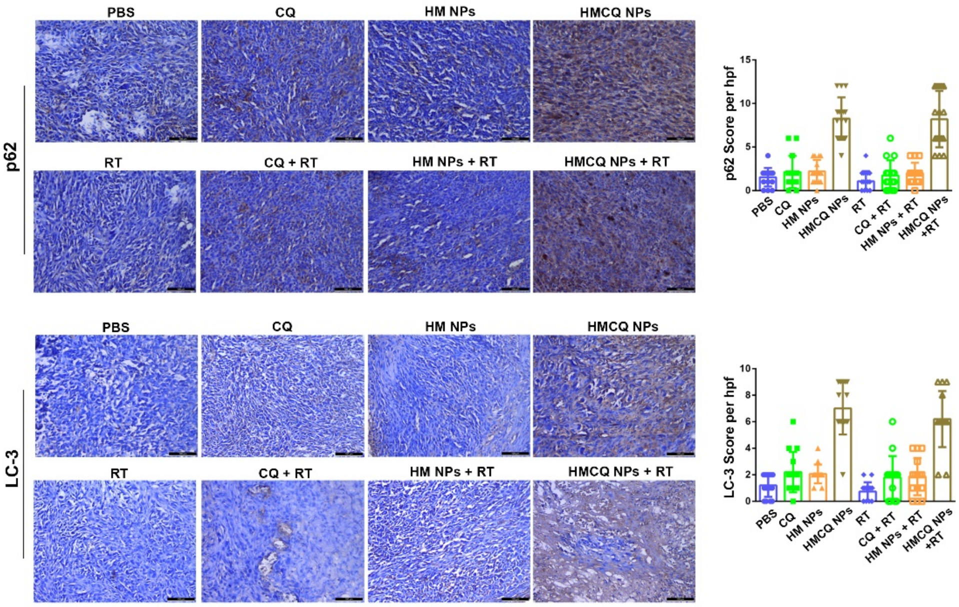

Figure S8. Left column is immunohistochemical staining for p62 and LC3 in T24 tumors treated with PBS, CQ, HSA-MnO_2 NPs and HSA-MnO_2-CQ NPs in the presence or absence of radiation therapy. Right column is the corresponding quantification of p62 expression and LC3 expression in immunohistochemical staining images.

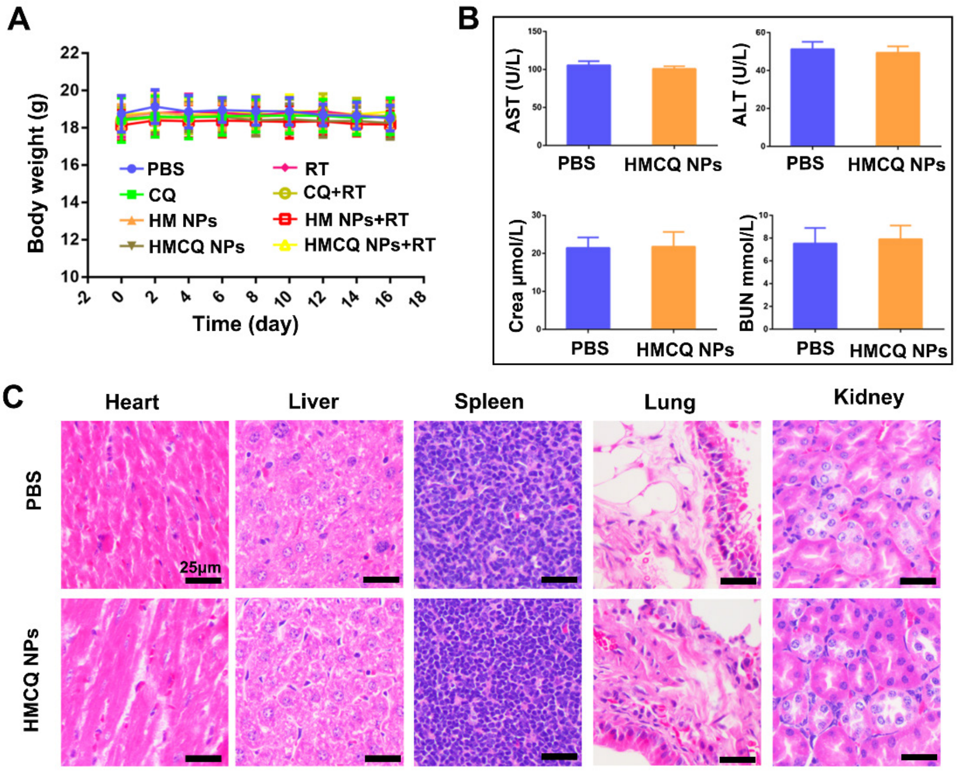

Figure S9. (A) Body weight of mice in different groups after treatments. (B) Hepatic

function evaluated by ALT and AST levels and renal function by UREA and CREA levels. (C) HE staining of the five organs (heart, liver, spleen, lung, and kidney).

Methods

Cellular uptake of CQ analyzed by High Performance Liquid Chromatography (HPLC)

To investigate the cellular uptake of CQ by T25 cells in acid condition or neutral condition, cells were plated in RPMI-1640 at pH 7.4. Next day, the medium was replaced with media at different pH (7.4 and 6.5). After 24 h of exposure to media at different pH, cells were treated with PBS, CQ (72 μM), HSA-MnO_2 NPs (180 μM) and HSA-MnO_2-CQ NPs (CQ 72 μM, MnO_2 180 μM) for 12 h and then collected. Cells were ultrasonicated with an ultrasonic cell disruption system (Biosafer250-88). H_2O_2 (100 μM) was added into mixture to avoid disturbance of MnO_2. After centrifugation, supernatant was analyzed using HPLC system equipped with C18 column. The elution solvent consisted of 1% diethylamine, acetonitrile and methanol at the ratio of 20:55:25 (v:v:v). The chromatographic analysis was operated at 25°C. Aliquots of 200 μL samples or standard solutions were injected onto the column with an elution buffer at flow rate of 1.0 ml/min.

O_2 assay in living cells in vitro

The effect of HSA-MnO_2-CQ NPs on degrading H_2O_2 into O_2 was evaluated in T24 cells under hypoxic condition using an oxygen probe (O_2 Microsensor, Unisense, Denmark). T24 cells were seeded onto plates in standard RPMI-1640 and covered with paraffin for 6 hours to provide a hypoxic environment. Then, the oxygen probe was inserted into the cell medium, HSA-MnO_2-CQ NPs (MnO_2 90 μM) were added, and the oxygen concentrations of the medium were recorded. The H_2O_2 concentration of cell medium under 6 hours hypoxic environment also evaluated by PeroXOquant assay kit (Pierce, USA). Result showed the H_2O_2 concentration of cell medium released by T24 hypoxic cells was 241.6 ± 49.5 μM, and significant amounts of oxygen were generated by the NPs reacting with H_2O_2 released by the cancer cells, thus producing oxygen in situ.

The blood circulation, tumor accumulation and biodistribution of HSA-MnO_2-CQ NPs.

The biodistribution of HSA-MnO_2-CQ NPs is performed by measuring CQ content in tumor and major organs. T24 Tumor bearing mice were intravenously injected with HSA-MnO_2-CQ NPs (CQ 40 μmol/kg, MnO_2 100 μmol/kg), tumor tissues and major organs were collected at 3, 6, 12, 24, and 48 h, the concentrations of CQ were analyzed by high performance liquid chromatography (HPLC).

The pharmaceutical analysis is performed by measuring CQ content in blood. T24 Tumor bearing mice were intravenously injected with HSA-MnO_2-CQ NPs (CQ 40 μmol/kg, MnO_2 100 μmol/kg) or free CQ (40 μmol/kg), blood samples were collected at 0.1, 0.5, 1, 2, 3, 6, 12 and 24 h, the concentrations of CQ in plasma were analyzed by HPLC.

MR imaging of HSA-MnO_2-CQ NPs *in Vivo*.

T24 tumors were developed by subcutaneously implanting $5×10^6$ T24 cells suspension in the lower back of mice. When the tumors were 150~200 mm^3, mice were

intravenously injected with HSA-MnO_2-CQ NPs (MnO_2 50 μmol/kg), then MRI was performed with a 7.0 T MRI scanner (BioSpec 70/20USR, Bruker, Germany). The T1-weighted MR images of bladder tumors were examined at 0, 12, 24 and 48 h after injection. MR scanning parameters were T1 propeller sequence, slice thickness 1 mm, TR/TE 789.8/10 ms, field of view 3.0 cm and matrix 256(a). The relative signal in-tensity (RSI) was calculated from the ratio of signals in the bladder tumor area to those in normal tissue areas.

The autophagy activity of T24 tumors after radiation therapy

To investigate the autophagy activity of T24 tumors after radiation therapy, T24 tumor bearing mice (tumor volume ~150mm^3) were intravenously injected with PBS, CQ (40μmol/kg), HSA-MnO_2 NPs (100μmol/kg) and HSA-MnO_2-CQ NPs (CQ 40μmol/kg, MnO_2 100μmol/kg) once per day for 4 days (day 0, day 1, day 2, day 3). For the radiation groups, X-ray radiation therapy was performed at day 4. The tumors were collected on day 6. For immunohistochemistry assay, tumor tissues were fixed in 10% formalin, paraffinized, and cut into 5 μm-thick sections. The slides were incubated with LC3 and p62 antibodies and then incubated with HRP-conjugated antibodies. The slides were visualized with a DAB Horseradish Peroxidase Color Development Kit, and counterstained with hematoxylin. The images were analyzed by Image-J software.

In vivo toxicity of HSA-MnO_2-CQ NPs.

To further assess the *in vivo* safety of HSA-MnO_2-CQ NPs, healthy Balb/c nude mice were intravenously injected with PBS or HSA-MnO_2-CQ NPs (CQ 40μmol/kg, MnO_2 100μmol/kg) once per day for 4 days. Blood samples and organs were harvested and analyzed at 24 h after treatments. From blood samples, alanine aminotransferase (ALT) and aspartate aminotransferase (AST) levels were used to evaluate hepatic function, renal function was evaluated by urea nitrogen (UREA) and creatinine (CREA) levels. The five organ tissues (heart, liver, spleen, lung, and kidney) were collected for HE staining.
